# The Role of Rosmarinic Acid in Alzheimer's Disease: A Promising Therapeutic Approach

**DOI:** 10.1002/alz70859_100764

**Published:** 2025-12-25

**Authors:** Sana Praween, Sukriti Vishwas

**Affiliations:** ^1^ School of Pharmaceutical Sciences, Lovely Professional University, Jalandhar, Punjab, Jalandhar, Punjab India; ^2^ School of Pharmaceutical Sciences, Lovely Professional University, Jalandhar, Punjab India

## Abstract

**Background:**

Alzheimer's disease (AD) is a progressive neurodegenerative disease characterized by cognitive decline, memory loss, and behavioral changes due to degeneration of cholinergic neurons in the cerebral cortex and hippocampus part of the brain. The underlying mechanisms involve oxidative stress, neuroinflammation, and the accumulation of amyloid‐beta plaques. As current treatments offer limited efficacy, exploring alternative therapeutic agents has become a key area of research. Rosmarinic acid (RA), a polyphenolic compound found in various herbs, has gained attention for its antioxidant, anti‐inflammatory, anti‐amyloid, and anti‐apoptosis properties, which may offer potential benefits in the treatment of AD.

**Method:**

This review compiles findings from preclinical and clinical studies investigating the effects of RA for the treatment of AD. Studies were identified through database searches and included both in vitro (cellular) and in vivo (animal) models of AD, as well as clinical trials examining RA's impact on cognitive function, neuroinflammation, and oxidative stress.

**Result:**

Experimental evidence suggests that RA exhibits anti‐inflammatory, antioxidant, and neuroprotective effects that could help mitigate the hallmarks of AD. In animal models, RA has been shown to reduce amyloid‐beta plaque deposition, decrease oxidative stress, and improve cognitive performance. Clinical studies, although limited, have reported positive trends in cognitive improvement and symptom management following RA supplementation, suggesting its potential as a supportive treatment.

**Conclusion:**

RA demonstrates significant promise as a therapeutic agent for AD. Its multifaceted mechanisms of action, including anti‐inflammatory, antioxidant, and neuroprotective properties, support its potential role in mitigating the pathological features of AD. Further clinical trials are needed to establish its efficacy and optimal use in AD management, but RA could offer a novel adjunct to existing treatments in combating this devastating disorder.

